# Multidisciplinary Care of Incidental Small Renal Masses After Cancer Remission

**DOI:** 10.7759/cureus.80857

**Published:** 2025-03-19

**Authors:** Vivie Tran, Megan T Mai, Sai Kodam, Luis Brandi, Simeon Jaggernauth

**Affiliations:** 1 Internal Medicine, Texas Tech University Health Sciences Center, Lubbock, USA; 2 Medical School, Texas Tech University Health Sciences Center, Lubbock, USA; 3 Internal Medicine, Texas Tech University Health Sciences Center El Paso, El Paso, USA; 4 Pathology, Texas Tech University Health Sciences Center, Lubbock, USA; 5 Oncology, University Medical Center Health System, Lubbock, USA

**Keywords:** bilateral nephrectomy, diffuse large b lymphoma, multidisciplinary decision-making, radiation & medical oncology, renal cell carcinoma (rcc)

## Abstract

This case report presents a 72-year-old male with a history of diffuse large B-cell lymphoma (DLBCL) who was found to have a 2.6 cm left renal mass during surveillance imaging. The clinical challenge lay in distinguishing between a recurrence of DLBCL and a new primary renal cell carcinoma (RCC), as both diseases are treated with different paradigms. A biopsy confirmed clear cell RCC, and the patient underwent a left nephrectomy with no evidence of metastasis or recurrence of lymphoma. This case highlights the importance of a biopsy in clarifying the diagnosis of renal masses, particularly in patients with prior malignancies. It also explores the potential role of radiation therapy as an alternative treatment option in cases where surgery may pose significant risks. A more comprehensive multidisciplinary approach would optimize patient outcomes and ensure individualized care.

## Introduction

Renal cell carcinoma (RCC) is a common malignancy, accounting for approximately 85% of all kidney cancers and 2% to 3% of all cancers. It is often diagnosed incidentally during imaging for unrelated conditions. While many small renal masses suspected of being RCC are treated by surgical resection or active surveillance without a biopsy, the clinical approach becomes more complicated when the patient has a history of prior malignancy. In such cases, a thorough diagnostic workup is essential to avoid misdiagnosis and ensure appropriate treatment [1].

Diffuse large B-cell lymphoma (DLBCL) is the most common type of non-Hodgkin lymphoma, characterized by aggressive behavior and a propensity for recurrence. In patients with a history of DLBCL, the development of a new mass can present a significant diagnostic challenge, as it may represent a recurrence of lymphoma or an entirely new primary malignancy such as RCC [2].

In this report, we present the case of a small renal mass found on follow-up imaging for post-treatment surveillance of DLBCL. The clinical dilemma centers on distinguishing between a possible recurrence of DLBCL and a new primary malignancy, as each diagnosis carries different treatment pathways.

## Case presentation

The patient is a 72-year-old male with a medical history significant for class II obesity, hypertension, type 2 diabetes mellitus, obstructive sleep apnea on continuous positive airway pressure, steatohepatitis (Brunt necroinflammatory grade 3, fibrosis stage III), and laparoscopic sleeve gastrectomy for weight loss. He initially presented with recurrent sinus infections, which were treated with multiple courses of oral antibiotics by his primary care physician and allergy specialist. Five months later, the patient was admitted to the hospital for shortness of breath and a foul odor emanating from his mouth and nasal cavity. He was diagnosed with community-acquired pneumonia. Imaging, including a CT of the neck, chest, and head, demonstrated a mass involving the right nasopharynx, suspicious for malignancy.

**Figure 1:**
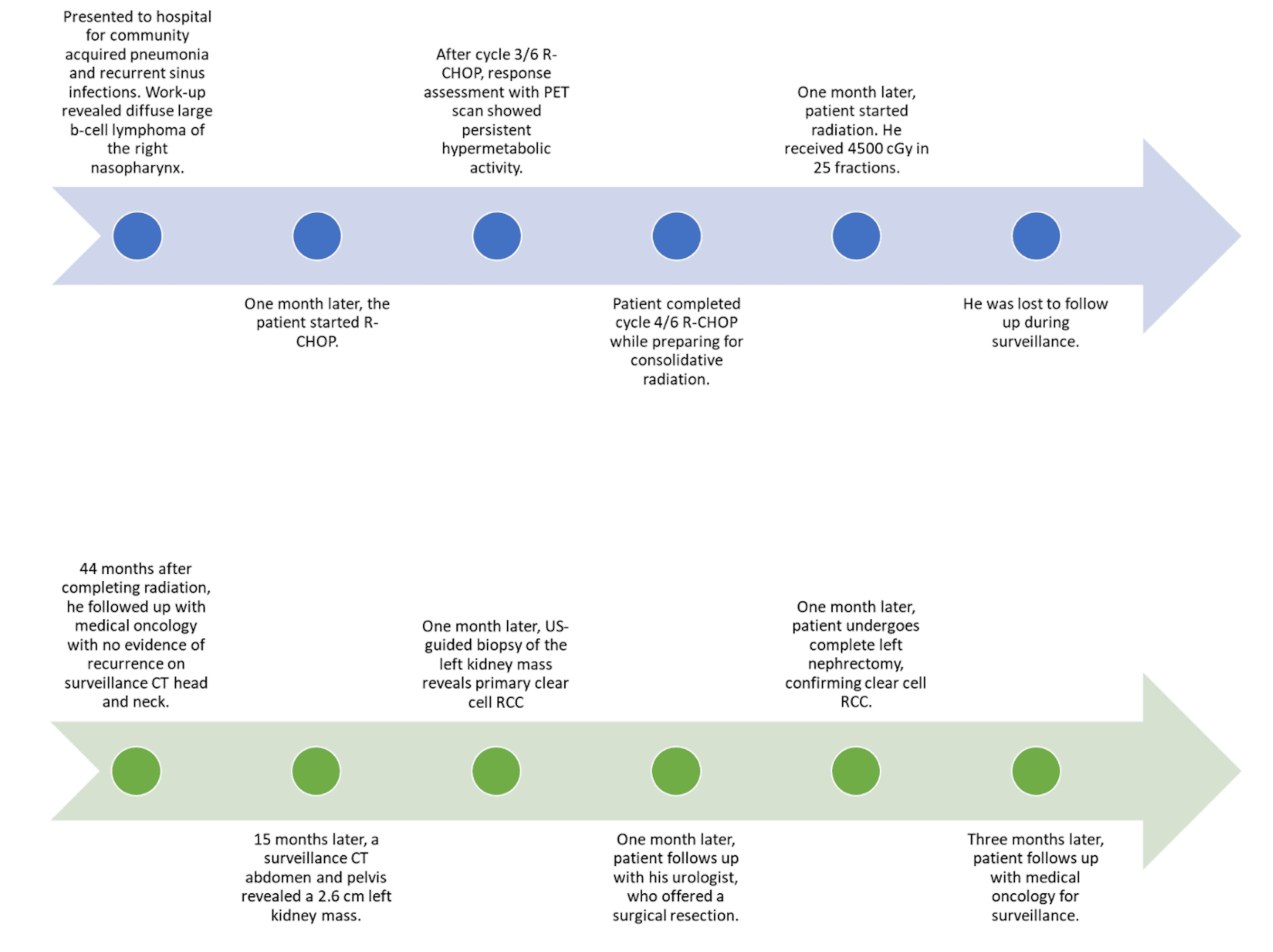
Case presentation timeline.

A subsequent biopsy of the nasopharyngeal mass confirmed high-grade B-cell lymphoma. The disease was staged as Ann Arbor stage IE, as it was confined to a single extranodal site (the nasopharynx) without evidence of nodal involvement or distant spread. Under the Ann Arbor system, stage I disease is limited to a single lymph node region (I) or a localized extranodal site (IE), distinguishing it from higher stages that involve multiple lymph node regions (stage II), both sides of the diaphragm (stage III), or disseminated extranodal involvement (stage IV) [3]. Given this staging, the patient was evaluated by Hematology/Oncology and was planned for rituximab-cyclophosphamide-hydroxydaunorubicin-oncovin-prednisone (R-CHOP) chemotherapy with prophylactic intrathecal methotrexate. Chemotherapy began one month after diagnosis, with a plan for six cycles of R-CHOP (Figure 1).

The patient tolerated chemotherapy well, with mild to moderate fatigue but no major complications. A response assessment after cycle 3 with a PET scan showed persistent right nasopharyngeal thickening with associated metabolic activity, but no evidence of metastasis was identified. Due to significant residual disease discussed with a multidisciplinary team, treatment escalation with consolidative radiation was recommended. He completed 4/6 cycles of R-CHOP and four doses of intrathecal methotrexate within two months of starting systemic therapy.

Due to the minimal response to systemic therapy, radiation therapy to the right nasopharynx was recommended and completed one month after cycle 4 of R-CHOP with a total dose of 4500 cGy in 25 fractions. His post-treatment course was uneventful, and he maintained good functional status, though he experienced aguesia and decreased appetite, which improved shortly afterward. He was lost to follow-up without surveillance imaging.

**Figure 2:**
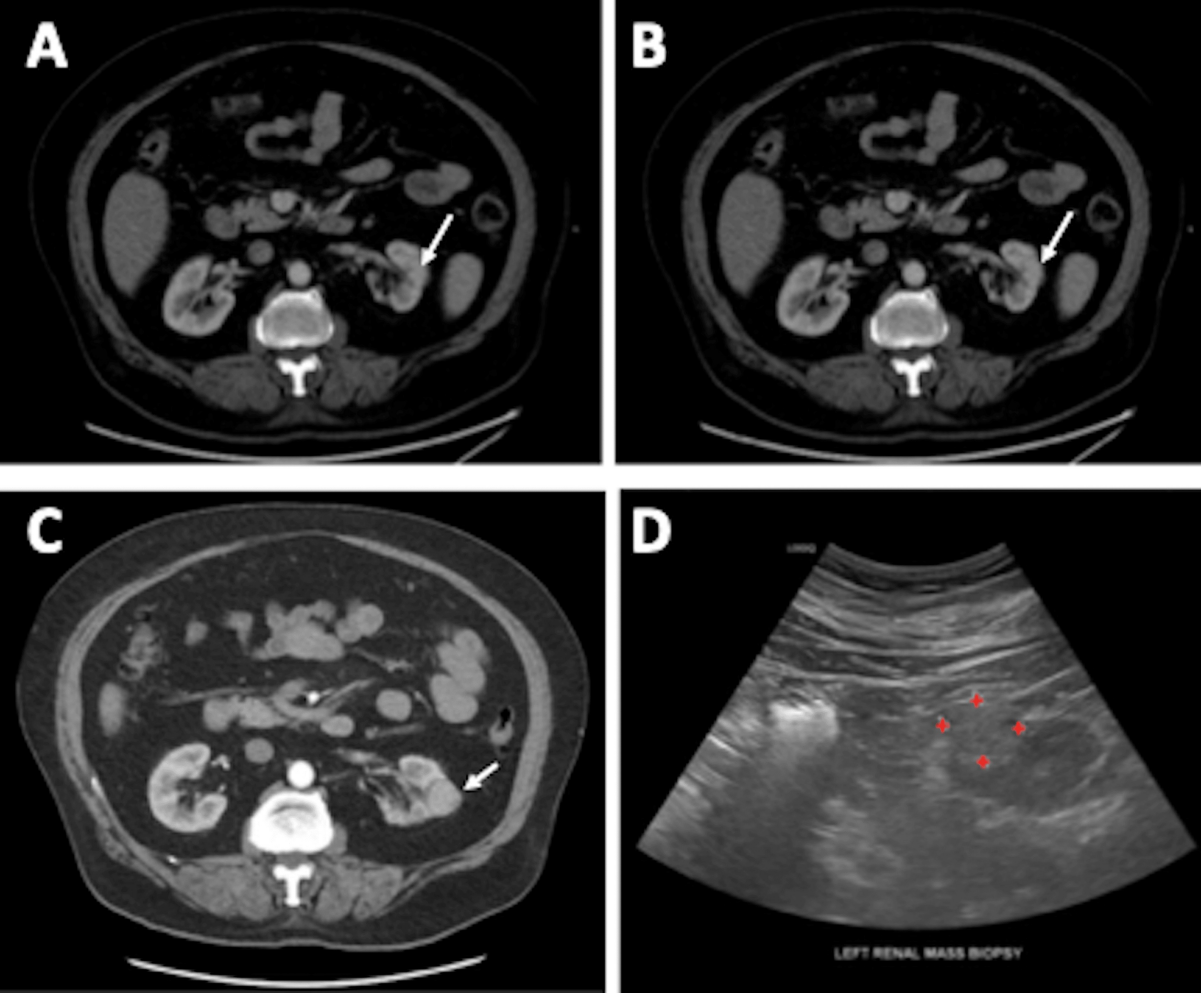
Radiographic development of left renal mass: a) Pre-R-CHOP CT chest/abdomen/pelvis, b) Response assessment PET/CT after cycle 3 R-CHOP, c) Surveillance CT chest/abdomen/pelvis showing renal mass, 59 months post-radiation, d) US-guided core biopsy of the left renal mass. R-CHOP: Rituximab, Cyclophosphamide, Hydroxydaunorubicin (Doxorubicin), Oncovin (Vincristine), Prednisone; PET/CT: Positron Emission Tomography/Computed Tomography.

At 44 months post-radiation, the first surveillance CT of the head and neck showed treatment-related changes without evidence of recurrence. However, no further surveillance imaging was done. At 59 months post-radiation, a CT scan of the abdomen and pelvis was ordered for surveillance and revealed a 2.6 cm mass in the left kidney (Figure 2).

**Figure 3:**
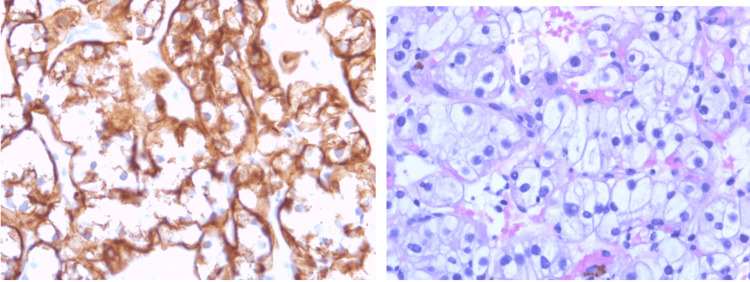
Pathology from ultrasound-guided left renal biopsy.

Subsequently, an ultrasound-guided biopsy was ordered to confirm the etiology of the small renal mass. The final pathology revealed RCC, clear cell subtype, Fuhrman grade 2 (PanCK positive, Pax8 positive, CD10 positive, CA-IX positive, CK7 negative, CD117 negative, and CD20 negative) (Figure 3).

The patient preferred to follow up with his established urologist for treatment recommendations on his newly diagnosed RCC. After discussing with his urologist from an outside institution, the patient decided to proceed with surgical management. He underwent a complete left nephrectomy 2 months after the biopsy at an outside institution. The final surgical pathology confirmed a T1a (3.5 cm) clear cell RCC. The patient followed up postoperatively with medical oncology with no clinical evidence of lymphoma recurrence. In the setting of early-stage RCC without an indication for adjuvant immunotherapy, the patient proceeded with post-operative surveillance.

Throughout his treatment, the patient has remained in good spirits, with no major complaints apart from mild fatigue. His most recent medical oncology follow-up for DLBCL and RCC showed no clinical evidence of DLBCL or RCC recurrence. Due to favorable RCC pathology, a CT chest/abdomen/pelvis at 1-year post-op was deemed appropriate for RCC surveillance, in addition to yearly history and physical for follow-up. Since the patient is more than two years out from definitive treatment for DLBCL, further surveillance with imaging is only done as clinically indicated.

## Discussion

Managing small renal masses poses a significant clinical dilemma, especially considering how biopsy results may influence disease management. In this case, we evaluated a 72-year-old male with a history of high-grade DLBCL who developed an enhancing left renal mass. The differential diagnosis included relapsed DLBCL and a new primary RCC, each requiring distinct treatment approaches with unique prognostic implications. However, due to the history of extranodal DLBCL, a biopsy was crucial in this context, as it provided clarity, guided management, and mitigated the risk of misdiagnosis and mistreatment. In many instances, RCC can be diagnosed and treated without a biopsy, particularly when imaging characteristics highly suggest RCC and no other pathology is suspected. However, the differential diagnosis of a new small renal mass for a patient with a history of DLBCL includes lymphoma recurrence, new primary RCC, and benign renal lesions, emphasizing the importance of a thorough diagnostic workup. The biopsy confirmed clear cell RCC, leading to a reassessment of treatment options, as systemic therapy for relapsed DLBCL differs significantly from the local therapy approach typically recommended for early-stage RCC [4].

While radical nephrectomy is a standard treatment for localized RCC, it may not always be the most appropriate choice [5]. In patients with significant comorbidities or those who have undergone prior extensive treatments, less invasive methods should be considered [6]. Although percutaneous thermal ablation is a less invasive treatment option, ASCO recommends its use for select tumors such that complete ablation will be achieved. Most studies on percutaneous thermal ablation are observational and retrospective [1]. To date, no major clinical trials have studied percutaneous thermal ablation for early-stage RCC. Alternatively, radiation oncology offers a valuable non-invasive treatment option, particularly when surgery carries significant risks or when patients are not surgical candidates. Radiation therapy, including stereotactic body radiation therapy (SBRT), can effectively control localized tumors while preserving kidney function and minimizing surgical complications [7].

Nephrectomy offers the benefit of complete tumor removal, accurate staging, and excellent long-term survival. Its definitive oncological control significantly reduces the risk of local recurrence. Additionally, the histopathological examination afforded by nephrectomy is critical for confirming the diagnosis and guiding further management. However, nephrectomy carries substantial risks, particularly for older patients or those with multiple comorbidities. Complications such as bleeding, infection, and extended recovery times are common, and nephrectomy reduces overall renal function, which can be problematic for patients already at risk for chronic kidney disease [8].

In contrast, SBRT offers a non-invasive, kidney-sparing alternative, especially suitable for patients who are not candidates for surgery due to age or comorbidities. SBRT delivers high-dose, targeted radiation to treat localized tumors while minimizing damage to surrounding tissue, offering effective tumor control with fewer acute side effects and a quicker recovery than surgery [7]. Notably, in the FASTRACK II trial, 33% of tumors were T1a. In the IROCK study, the overall greatest tumor dimension averaged 43.6 ± 27 mm, while those who specifically received 25 Gy in one fraction had an average greatest tumor dimension of 37.1 ± 10.6 mm. While the FASTRACK II trial and IROCK studies showed promising outcomes and local control, the long-term oncological outcomes are less well-established compared to nephrectomy [9-11]. For aggressive or larger tumors (greater than 10 cm), nephrectomy may be a safer treatment option, considering the risks associated with expanded radiation volume coverage. Additionally, nephrectomy allows for the histopathological assessment of the surgical specimen, which most accurately confirms the diagnosis and staging.

The choice between nephrectomy and SBRT should be based on the patient's overall health, tumor characteristics, and personal preferences. In younger, healthier patients with localized RCC, nephrectomy remains the first-line treatment. However, in older patients or those with significant comorbidities, SBRT offers a viable, less invasive alternative that can effectively manage localized disease while minimizing surgical risks.

## Conclusions

This case highlights the importance of individualized treatment planning for patients with complex medical histories. Although nephrectomy was the chosen approach following the biopsy diagnosis of clear cell RCC, consideration of alternative treatments, such as SBRT, might have offered benefits in terms of preserving renal function and minimizing surgical risks.

The integration of multidisciplinary expertise, particularly from radiation oncology, offers valuable alternatives that may preserve renal function and minimize surgical risks. As the field of oncology continues to evolve, clinicians must remain vigilant in exploring all available treatment modalities, ensuring that patient management strategies are tailored to optimize outcomes and align with the patient's values and preferences. This case serves as a reminder of the complexities inherent in oncological care and the need for a collaborative, patient-centered approach to decision-making.
